# Description of a new species of the genus *Laelaspis* Berlese (Acari, Mesostigmata, Laelapidae) from Iran

**DOI:** 10.3897/zookeys.549.7435

**Published:** 2016-01-06

**Authors:** Shahrooz Kazemi, Nazanin Mehrzad, Malihe Latifi

**Affiliations:** 1 Department of Biodiversity, Institute of Science and High Technology and Environmental Sciences, Graduate University of Advanced Technology, Kerman, Iran; 2 Department of Plant Protection, Faculty of Agriculture, Vali-e-Asr University of Rafsanjan, Rafsanjan, Iran

**Keywords:** Parasitiformes, Dermanyssoidea, taxonomy, myrmecophilus mites

## Abstract

A new species of the genus *Laelaspis* Berlese, *Laelaspis elongatus* **sp. n.** is described based on adult female and male specimens collected in association with *Pheidole pallidula* (Nylander) (Hym., Formicidae) in Ahwaz, Khuzestan Province, southwestern Iran, and also Acinopus (Acinopus) picipes (Olivier) (Col., Carabidae) in Bam, Kerman Province, southeastern Iran.

## Introduction

*Laelaspis* (Mesostigmata: Laelapidae) was originally established by Berlese (1903) as a subgenus of *Laelaps* Koch, 1939, with the type species *Laelaps astronomicus* Koch, 1839, and was later elevated to genus by Berlese (1920, 1924). Although Vitzthum (1943) considered *Laelaspis* as a subgenus of *Hypoaspis* Canestrini, 1885 sensu lato, and this idea was followed by some subsequent authors (e.g. Evans and Till 1966, Hunter and Glover 1968, Van Aswegen and Loots 1970, Lapina 1976, Bregetova 1977), Berlese’s (1920, 1924) classification is followed by more authors (e.g. Hunter 1961, 1964, Hunter and Davis 1962, Bai and Gu 1993, Joharchi et al. 2011, 2012a, b, Babaeian et al. 2013, Ramroodi et al. 2014, Kazemi 2015).

The original description of *Laelaspis* was very poor. Although Hunter (1961) reviewed the genus and some authors tried to clarify its boundaries since Hunter’s (1961) work (Evans and Till 1966, 1979, Bregetova 1977, Joharchi et al. 2011), the genus diagnosis was not clear. Recently, Kazemi (2015) reviewed the concept of *Laelaspis*, presented its generic diagnosis and detailed diagnosis, and considered the genus to include 40 described species of usually myrmecophilous mites, although some species occur in different habitats such as free-living in soil, litter and moss, or associated with beetles, and mammals or their nests (Berlese 1903, 1904, 1920, Evans and Till 1966, Lapina 1976, Hunter 1961, 1962, 1964, Hunter and Davis 1962, Hunter and Glover 1968, Joharchi et al. 2012a).

Until now 13 species of *Laelaspis* have been reported from Iran, including seven new species found mostly in association with ants, but also rarely in soil and litter (Faraji et al. 2008, Joharchi et al. 2012a, b, Babaeian et al. 2013, Kazemi and Rajaei 2013, Ramroodi et al. 2014, Kazemi 2015). Herein, we follow Kazemi’s (2015) diagnosis of the genus and describe a new species of *Laelaspis* from Iran.

## Material and methods

Mite specimens of the new species were removed from under elytra of a beetle host, Acinopus (Acinopus) picipes (Olivier, 1795) (Col., Carabidae) in Bam County, Kerman Province, southeastern Iran, and also associated with an ant host, *Pheidole pallidula* (Nylander) (Hymenoptera, Formicidae) in Ahwaz County, Khuzestan Province, southwestern Iran. Mite specimens were cleared in Nesbitt’s fluid and then mounted in Hoyer’s medium on microscope slides before examination.

Morphological observations, measurements and illustrations were made using compound microscopes (Olympus BX51) equipped with differential interference contrast and phase contrast optical systems, and a drawing tube. Figures were prepared using Microsoft Office Powerpoint 2003 based on scanned line drawings. Measurements were made in micrometers (μm). Dorsal shield length and width were respectively taken from the anterior to posterior shield margins along the midline, and from the lateral margins at the broadest level between setae *j6*-*J1*. The length of sternal shield was measured from the anterior to posterior margins of the shield along the midline, and its width at the lateral margins at the levels of setae *st2* and *st3*. The length of genitiventral shield was taken from the anterior margin of the hyaline extension to the posterior margin of the shield along the midline and also from the level of setae *st5* to the posterior tip of the shield; shield widths were taken at the level of *st5* and also at the broadest points. The anal shield length and width were measured along its midline from the anterior to posterior margins, including the cribrum, and at the broadest point, respectively. The leg lengths were taken dorsomedially from the base of the coxa to the apex of the tarsus, excluding the ambulacrum (stalk, claws and pulvillus). The length of the second cheliceral segment was measured from the base to the apex of the fixed digit, and its width at the broadest point. The length of the fixed cheliceral digit was taken from the dorsal poroid to the apex, and that of the movable digit from the base to apex. The notation for idiosomal setae follows that of Lindquist and Evans (1965) adapted by Evans and Till (1965, 1966) and Lindquist (1994), and that for leg and palp setae follows Evans (1963a, 1963b). The notation for idiosomal pore-like structures as gland pores and poroids follows mostly Athias-Henriot (1971, 1975), adapted by Kazemi et al. (2014).

Kazemi (2015) indicated in the diagnosis of the genus *Laelaspis* that the genitiventral shield bears at least two pairs of setae on its lateral margins, always including *st5* and *JV1*. Herein, based on Evans and Till (1965) and also Lindquist (1994), we consider that *st5* and *ZV1*, not *JV1*, are the two ‘core’ setae always inserted on the genitiventral shield of *Laelaspis* species, and that the arrangement of opisthogastric setae in members of the genus is similar to that of the new species illustrated herein.

## Taxonomy

### Genus Laelaspis

Taxon classificationAnimaliaMesostigmataLaelapidae

Berlese, 1903

#### Type species.

*Laelaps astronomicus* Koch, 1839

#### Diagnosis.

The genus diagnosis of Kazemi (2015) was followed.

### Laelaspis elongatus sp. n.

Taxon classificationAnimaliaMesostigmataLaelapidae

http://zoobank.org/FC07A0CF-4110-4DEC-A180-F5A9C1B781C8

Figures 1–4 , 5–8 , 9–14

#### Diagnosis (adult male and female).

Dorsal shield relatively elongate, ratio of length/width of shield≈1.7, with 39 pairs of setae, including *Px2*–*3*, and three unpaired setae *Jx*, setae mostly subequal in length and relatively short, usually not reaching to following seta base, *j1* and *z1* shortest, subequal and lanceolate, situated subventrally, *J5* and *Z5* short, ratio of *J5*/*Z5* length≈1.5. Sternal shield of female with lineate-reticulate ornamentation on anterior and lateral surface, ratio of shield length/width (at *st3* level) ≈ 0.9; subequal sternal setae shorter than distance to following seta base. Genitiventral shield of female longer than wide, ratio of length/width (at broadest level) ≈ 1.9, shield bearing two pairs of smooth setae, *st5* and *ZV1*. Anal shield almost as long as wide (slightly wider than long in specimen removed from beetle host); circumanal setae smooth, postanal seta slightly shorter than para-anals. Opisthosomal membrane in female with 17 pairs of setae, 13 pairs in male. Peritrematal shields well developed, hind edge of shield not reaching to posterior edge of subtriangular parapodals. Peritremes long, reaching to anterior level of coxae I. Epistome with smooth and subtriangular anterior margin. Subcapitular setae *h3*>*h1*>*cs*>*h2*. Movable and fixed cheliceral digits in female bidentate; fixed and movable digits of male chelicera each with an acuminate weakly sclerotised apical projections, fixed digit with a prominent tooth (apical hook), movable digit thickened, unidentate, spermadactyle finger-like, slightly shorter than movable digit, parallel with an acuminate protrusion inserted below it. Leg chaetotaxy normal for genus, including nine setae on genua IV and 10 setae on tibiae IV, setae mostly simple and slender or slightly thickened, except following setae: *pv1* on femur I, *pv* on genu I, *pv* and *pd2* on tibia I thickened; *pd2* on femur I short and spine-like. Seta *pv1* in femura II of male thicker than those in female.

#### Description.

**Figures 1–4.**
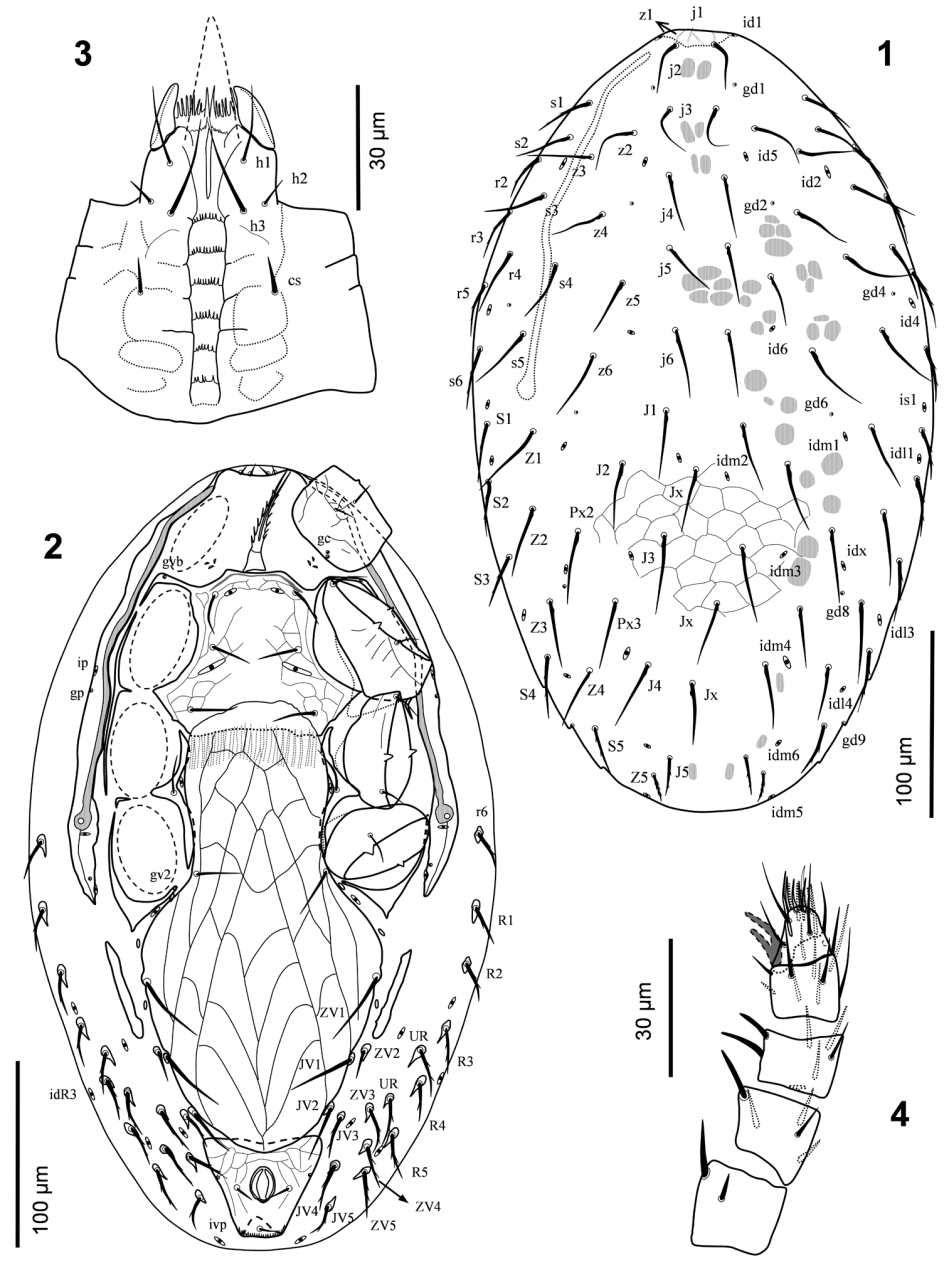
*Laelaspis elongatus* sp. n. Female: **1** dorsal idiosoma **2** ventral idiosoma **3** subcapitulum **4** palp.

***Female*** (n = 3). *Dorsal idiosoma* (Fig. 1). Dorsal shield 414–423 long, 243–248 wide, covered dorsal idiosoma completely, with lineate-reticulate ornamentation throughout, bearing 39 pairs of setae and three unpaired setae *Jx* between *J1* and *J5*, *j1*and *z1* subequal, lance-like, shortest (9–10), situated subventrally, *J5* (23–24) and *Z5* (17–18) shorter than the rest of setae, *J5* sparsely barbed, *Z5* pilose, other dorsal setae subequal, 25–43 long, usually with a small enlargement near base, setae *s1*, *s6*, *S1*–*5*, *r2*–*5*, with few sparse barbs. Dorsal shield with 16 pairs of poroids (oval-shaped symbols) and six pairs of gland pores, *gd1*–*2*, *gd4*, *gd6*, *gd8*–*9* (circular symbols).

*Ventral idiosoma* (Fig. 2). Tritosternum with a short columnar base, 7–8 long, 10 wide at base, 5–6 wide at apex, and two pilose laciniae, free for 43–46 μm, fused 7 μm. Soft integument behind coxae I with three pairs of gland openings flanked by two minute valves. Sternal shield 84–87 long, 62–66 wide at *st2* level, 88–98 at *st3* level, distinctly reticulate on anterior and lateral surface; anterior margin of shield bilobed, posterior margin slightly concave; anterolateral corners narrowly fused to endopodal platelets between coxae I-II, bearing gland pores *gvb*, and fused to exopodals behind coxae II; shield with three pairs of smooth setae, *st1*–*3* (22–27) and two pairs of slit-like poroids, *iv1* between setae *st1*, and *iv2* enlarged, behind setae *st2*. Metasternal setae (25–26) on free endopodal platelets between coxae II-III, poroids *iv3* on soft cuticle. Genitiventral shield elongate, 226–238 long from anterior to posterior margins, 139–153 long from *st5* level to posterior edge, 71–75 wide at *st5* level, 119–128 wide at broadest point anteriad to *ZV1* level; anterior hyaline margin irregularly convex, covering part of posterior smooth area of sternal shield reaching to *st3* level, shield gradually narrowed from widest point, posteriorly convex, occasionally slightly bluntly tapered, and covered anterior margin of anal shield, inner Λ-shaped lines flanked nine cells; setae *st5* (25–27) and *ZV1* (42–44) inserted on lateral margins of shield. Anal shield subtriangular, anterior margin of shield slightly concave, 59–61 long, 59–66 wide, lineate-reticulate on anterior and lateral surface; circumanal setae smooth, fine, para-anal setae (13–14) slightly longer than postanal seta (10–11); cribrum developed posteriorly; para-anal gland pores *gv3* on lateral shield margins at level of anterior edge of anal opening. Peritrematal shields well developed along peritremes, anteriorly narrowed and fused to dorsal shield behind setae *z1*, bearing two pairs of pore-like structures near external margin of shield, a pair of gland pores at level of anterior edge of coxae III and one pair of poroids at level of posterior edge of coxae II; poststigmatic area with a longitudinal line from stigmata to shields hind edge, and with two pairs of poroids and one pair of gland pores. Peritremes long (205–208), reaching anterior margin of coxae I. Exopodal platelet between coxae II-III narrowly developed, exopodal between coxae III-IV small, parapodals developed and subtriangular posteriorly, bearing *gv2*. Opisthogastric soft integument bearing a pair of primary metapodal platelets narrowly elongate, laterad of genitiventral shield, 51–54 long, 6–7 wide; two pairs of paragenital minute platelets between primary metapodals and genitiventral shield and one pair between parapodal and ginitiventral shields; seven pairs of poroids including *iv5*, *ivp* and *idR3*, and 17 pairs of setae, *JV1* (35–41), *JV2* (30–33), *ZV2* (19–22) smooth, others barbed, 18–28 long.

**Figures 5–8.**
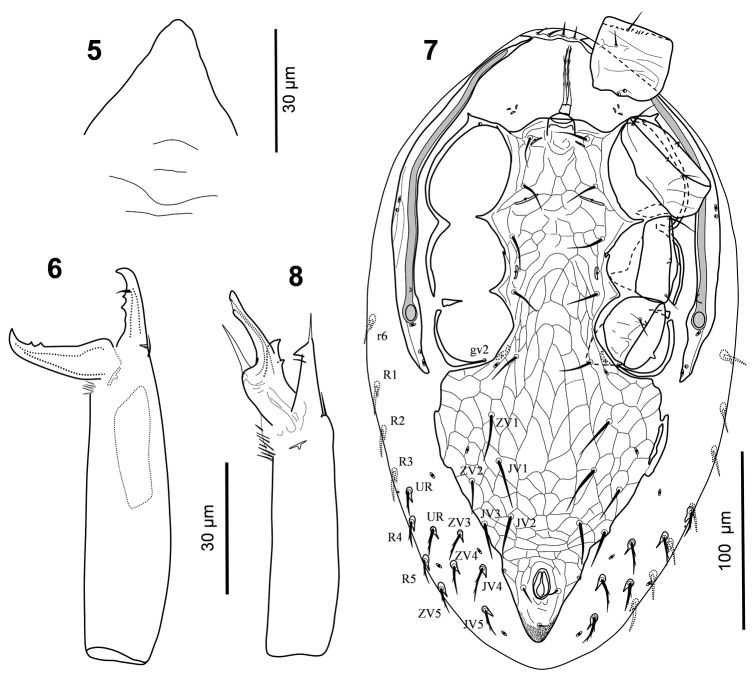
*Laelaspis elongatus* sp. n. **5–6** Female: **5** epistome **6** chelicera; **7–8** Male: **7** ventral idiosoma **8** chelicera.

*Gnathosoma* (Figs 3–4, 5–6). Anterior margin of epistome subtriangular, smooth (Fig. 5). Corniculi horn-like, 45–47 long. Salivary stylets narrow and apically pointed, aligned beneath corniculi. Internal malae fringed, with a pair of smooth adjacent median projections, flanked by shorter and thinner lateral projections. Labrum acuminate, pilose, considerably longer than internal malae and corniculi. Hypostomal and capitular setae smooth, *h3* (21–23)>*h1* (17–18)>*cs* (9–10)>*h2* (7–8). Deutosternal groove with six rows of denticles, three basal rows narrower, with 3–7 denticles, rest anterior rows slightly wider, with 5–11 denticles (Fig. 3). Second segment of chelicera 86–88 long, 18–19 wide; fixed digit of chelicera 17–18 long, movable digit 23–25 long, both digits bidentate; dorsal seta short and setiform (Fig. 6). Palp 70–73 long; palp chaetotaxy normal for Laelapidae with 2, 5, 6, 14, 15 setae on trochanter, femur, genu, tibia and tarsus, respectively , all setae smooth, *al1* and *al2* on palpgenu and *al* on palpfemur slightly thickened and subspatulate, *v1* on palptrochanter slightly thickened, somewhat spine-like; palptarsus apotele two-tined, basal tine shorter (Fig. 4).

**Figures 9–14.**
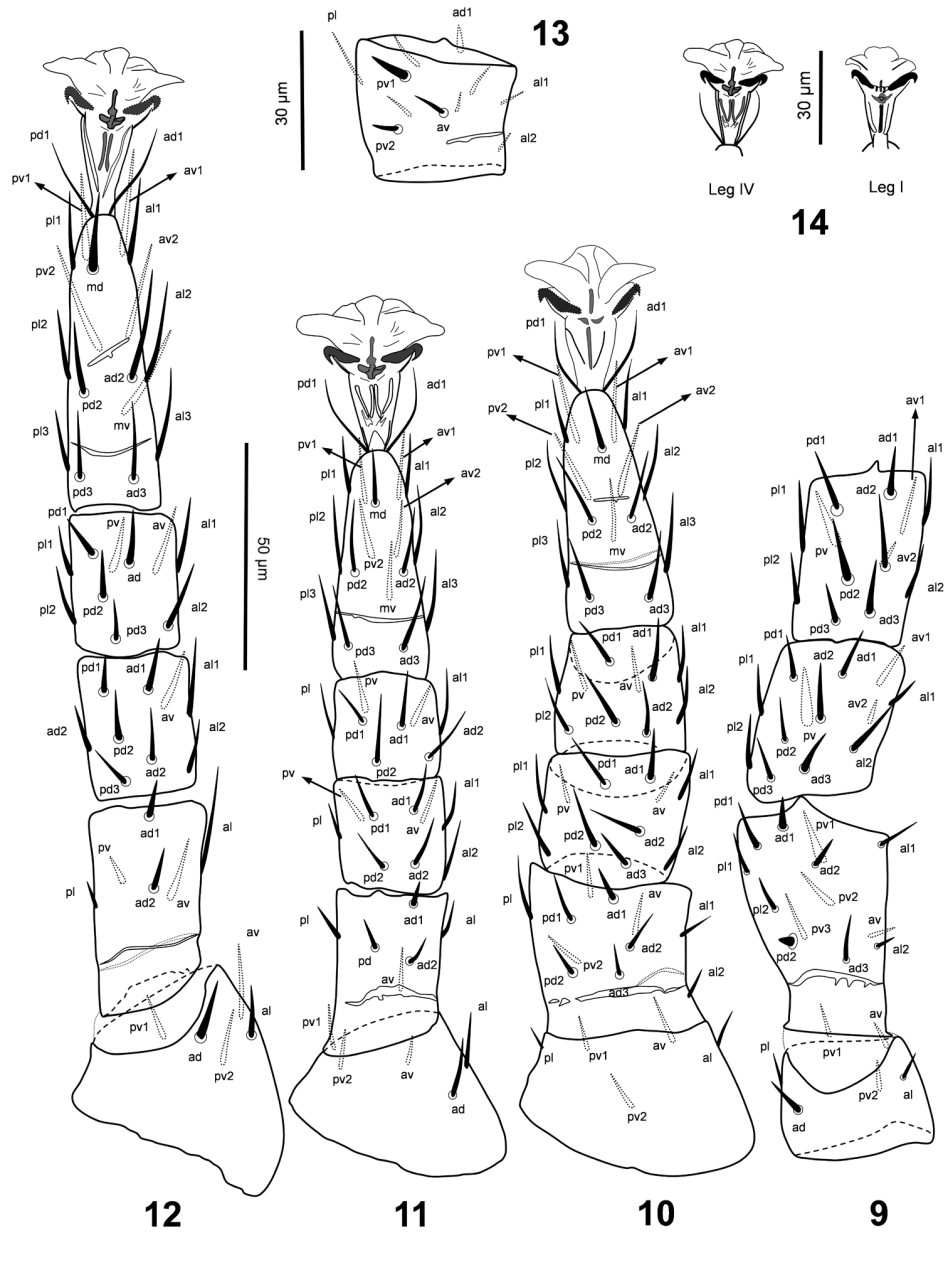
*Laelaspis elongatus* sp. n. Female: **9–12** legs I-IV, dorsal view; **13** Male: femur II, ventral view **14** Female, ambulacra I and IV.

*Legs* (Figs 9–12, 14). Leg chaetotaxy normal for Laelapidae (sensu Evans and Till 1965). Ambulacra of legs I-IV subequal, 32–35 long, claws and pulvilli developed, ambulacral stalk broad. Lengths of legs I-IV 267–278, 190–192, 180–183 and 207–212, respectively. Lengths of femora I 70–73, II 32–33, III 31–34, IV 43–46; genua I 33–36, II 28–31, III 19–22, IV 28–31; tibiae I 38–39, II 25–26, III 21–24, IV 28–31; tarsi I 82–83, II 50–54, III 48–52, IV 65–68. Leg setae mostly narrow, needle-like and moderately short, as diagnosis of species. Coxa I bearing two gland pores (*gc*) (Fig. 2).

***Male*** (n = 1). *Dorsal idiosoma*. Idiosoma 345 long, 205 wide. Dorsal shield 326 long, 195 wide, covering most of dorsal idiosoma but leaving a narrow exposed band of soft cuticle laterally and posteriorly; length of setae: *j1* 8, *z1* 7, *J5* 20, *Z5* 15, others 23–34; other dorsal characters similar to those in female.

*Ventral idiosoma* (Fig. 7). Tritosternal base 8 long, 7 wide at base, 4 wide at apex, with two pilose laciniae, free for 26 μm, fused basally for 5 μm. Holoventral shield 291 long from anterior to posterior margins of shield, 59 wide at level of *st2*, 66 at *st3* level and 132 at broadest point (*ZV1* level), anterolateral edges of shield narrowly fused to endopodals between coxae I-II, including gland pores *gvb*, then fused to exopodals behind coxae II-III; shield surface lineate-reticulate throughout, with five pairs of subequal smooth sternal setae, *st1*–*5* (19–22), and five pairs of smooth ventral setae, *ZV1*, *JV1–2* (24–28), *ZV2*, *JV3* (20–21), plus three smooth circumanal setae, para-anals (11) longer than postanal seta (8), with five pairs of poroids, *iv1*–*3* slit-like, *iv2* enlarged, and with a pair of gland pores *gv3* on lateral margins of shield at anterior level of anal opening; cribrum developed; metapodal platelets narrow, completely or partly fused to shield. Soft integument with 13 pairs of subequal and barbed setae (14–18), inserted on small platelets, and five pairs of poroids, including *idR3*. Peritremes relatively narrow and long (172), reaching to anterior margin of coxae I. Peritrematal shields similar to those in female, poststigmatic region slightly longer, reaching to posterior level of parapodals.

*Gnathosoma* (Fig. 8). Epistome, subcapitulum and palp characters similar to those in female. Hypostomal and capitular setae smooth, *h3* (18)>*h1* (14)>*cs* (9)>*h2* (6). Corniculi 9 long. Second segment of chelicera 76 long, 15 wide; fixed digit 22 long, unidentate (apical hook), with an apical fine projection, pilus dentilis short and setiform; movable digit thick, 21 long, with one small tooth, spermatodactyl almost straight, slightly shorter (18) than movable digit, parallel to and longer than a narrow and apically fine projection below it (Fig. 8).

*Legs* (Fig. 13). Leg chaetotaxy and characters similar to those in female, except *pv1* in femura II thicker than same setae in female. Lengths of legs I-IV 234, 166, 154, 187, respectively. Lengths of femora I 37, II 29, III 26, IV 29; genua I 29, II 22, III 18, IV 23; tibiae I 29, II 18, III 18, IV 25; tarsi I 68, II 42, III 43, IV 46; ambulacra I-IV 28–32.

#### Material examined.

Holotype: female, southeastern Iran, Kerman Province, Bam County, under elytra of Acinopus (Acinopus) picipes (Olivier, 1795) (Col., Carabidae) (29°06'096"N; 58°18'866"E), 1107 m above sea level, 31 August 2011, coll. N. Mehrzad, deposited in Acarological Collection, Institute of Science and High Technology and Environmental Sciences, Graduate University of Advanced Technology, Kerman, Iran (ACISTE). Paratypes: two females and one male, southwestern Iran, Khuzestan Province, Ahwaz County, associated with *Pheidole pallidula* (Nylander, 1849) (Hym., Formicidae), 18 Nov. 2013, deposited in ACISTE.

#### Etymology.

The species epithet “*elongatus*” was chosen based on the elongated dorsal and genitiventral shields of the female.

#### Remarks.

The new species, *Laelaspis elongatus* sp. n., can be easily distinguished from other members of the genus by combination of three unique characters: (1) postanal seta slender and slightly shorter than para-anal setae (thicker and longer than para-anals in other described species, except *Laelaspis kamalii* Joharchi et al. 2012 with subequal in length circumanal setae which can be easily distinguished from the new species by several characters such as: edentate movable digit in *Laelaspis kamalii* [bidentate in *Laelaspis elongatus* sp. n.], setae *J5* and *Z5* similar in length in *Laelaspis kamalii* [ratio of *J5*/*Z5* length≈1.5 in *Laelaspis elongatus* sp. n.], posterior edge of peritrematal shields well past hind edge of parapodal shields in *Laelaspis kamalii* [posterior edge of peritrematal shields shorter than hind edge of parapodal shields in *Laelaspis elongatus* sp. n.], genitiventral shield in *Laelaspis kamalii* wide and almost trapezoidal [the shield narrower and posteriorly convex in *Laelaspis elongatus* sp. n.]); (2) length/width ratio of the genitiventral shield in the new species almost 1.9 (the ratio less than 1.6 in other described species); (3) male chelicera with pointed and fine projections on the fixed and movable cheliceral digits, as those in figure 8 (without these projections in other described species).

## Discussion

The genitiventral shield in *Laelaspis* species is often longer than wide, and the length/width ratio at the broadest level of the shield is usually between 1–1.5 (in *Laelaspis aviator* Berlese, 1920 and *Laelaspis volgini* Shereef and Afifi, 1980 wider than long), but in *Laelaspis elongatus* sp. n. this ratio is almost 1.9. On the other hand, in some keys (Joharchi et al. 2012a, Ramroodi et al. 2014) *Laelaspis secedens* Berlese, 1920 has been separated from related species by its elongated genitiventral shield. We studied the photographs taken from slide 201/22 (the holotype of *Laelaspis secedens*) in the Berlese’s collection in Florence, Italy, and also the measurements of the species: the body size length 432, width 288; the genitiventral shield length 211 and its width 141 in the broadest point (pers. comm. of R. Nanelli with senior author), giving a ratio of the length/width of the genitiventral shield in *Laelaspis secedens*≈1.5, a usual ratio within the genus. On the other hand, *Laelaspis elongatus* sp. n. can be easily distinguished from *Laelaspis secedens* by some more features like postanal seta in *Laelaspis elongatus* sp. n. fine and shorter than para-anals, but serrate, considerably thicker and longer than para-anals in the latter species, length ratio of Z5/*J5* in the new species ≈ 0.75, but Z5/*J5* ≈ 2 in *Laelaspis secedens*, *JV5* almost as half-length of *JV1* in *Laelaspis elongatus* sp. n., but *JV5* almost as long as *JV1* in *Laelaspis secedens*.

So far only two species of the genus have been reported in association with beetles, *Laelaspis aviator* and *Laelaspis secedens*. In this research, two female and one male specimens were found in association with an ant, *Pheidole pallidula*, in Ahwaz County (southwestern Iran), but one specimen of the new species was also found under the elytra of a carabid beetle in Bam County (southeastern Iran). This is the third report of a *Laelaspis* species from a beetle.

## Supplementary Material

XML Treatment for Laelaspis

XML Treatment for Laelaspis elongatus
